# Effect of pirfenidone on wound healing in lung transplant patients

**DOI:** 10.1186/s40248-018-0129-4

**Published:** 2018-06-14

**Authors:** Amber Mortensen, Lauren Cherrier, Rajat Walia

**Affiliations:** 1 Department of Pharmacy, St. Joseph’s Hospital and Medical Center, Phoenix, AZ USA; 2 Division of Pulmonology, Norton Thoracic Institute, St. Joseph’s Hospital and Medical Center, 500 W. Thomas Rd., Ste. 500, Phoenix, AZ USA

**Keywords:** Idiopathic pulmonary fibrosis/drug therapy, Idiopathic pulmonary fibrosis/surgery, Lung transplantation, Pirfenidone, Surgical wound dehiscence, Wound healing

## Abstract

**Background:**

The drug pirfenidone has been shown to slow the progression and decrease mortality of idiopathic pulmonary fibrosis (IPF). Its exact mechanism is unknown, but it likely inhibits pro-fibrotic cytokine transforming growth factor beta, a known contributor to wound healing. We evaluated whether patients taking pirfenidone until lung transplantation had increased risk of impaired wound healing post-transplant. This information could determine whether pirfenidone should be discontinued prior to listing to allow for a wash-out period.

**Methods:**

We retrospectively reviewed patients who underwent lung transplantation for pulmonary fibrosis at Norton Thoracic Institute in Phoenix, Arizona, from January 2014 to December 2015.

**Results:**

We describe 18 patients who took pirfenidone up to a month before transplant. Aside from one patient who experienced sternal dehiscence due to a surgical issue, all remaining patients did well with no evidence of airway dehiscence. Each of these 17 patients had been on pirfenidone for at least 30 days; nine patients had been on pirfenidone for over 90 days. Baseline characteristics including age, sex, body mass index, renal function, liver function, glucose level, pre-transplant corticosteroid use, and post-transplant immunosuppressant therapy were similar.

**Conclusions:**

In our experience, pirfenidone may be safely continued until lung transplantation. Only one patient in our series experienced impaired wound healing related to a surgical issue, even when pirfenidone was continued until lung transplantation. We found no evidence of impaired wound healing or airway complications after lung transplantation in patients who were treated with pirfenidone before lung transplantation.

## Introduction

Idiopathic pulmonary fibrosis (IPF) is a progressive disease involving the replacement of normal lung parenchyma with fibrotic tissue. Patients with IPF often experience difficulty breathing and persistent cough, and their condition may advance or become fatal within 3 to 5 years of diagnosis [1, 2]. In October 2014, the Food and Drug Administration approved the first two medications for treatment of IPF: pirfenidone (Esbriet^®^) and nintedanib (Ofev^®^). Pirfenidone has been shown to successfully slow the progression of IPF, and patients in pirfenidone clinical trials have shown stabilization of IPF and decreased IPF-related mortality [3, 4].

The exact mechanism of action of pirfenidone is unknown [5], but its anti-fibrotic effect is thought to result from inhibition of transforming growth factor beta (TGF-β), a pro-fibrotic cytokine [6]. Pirfenidone also decreases inflammation and has antioxidant effects [6]. In fibrotic diseases such as IPF, TGF-β is excessively produced, accumulating and replacing healthy tissue and leading to overproduction of fibroblasts [7]. Although it has been shown to improve or stabilize pulmonary function, pirfenidone does not cure the underlying disease—so diseases may still progress to an advanced stage [1, 8]. Current treatment guidelines for patients with advanced IPF strongly recommend lung transplantation, which has been shown to decrease mortality in patients with IPF by 75% [1, 9].

In addition to its pro-fibrotic properties, TGF-β is also known to play a significant role in all stages of wound healing [7], and therefore inhibition of TGF-β may result in impaired wound healing. Discontinuing pirfenidone to allow for a wash-out period pre-transplantation, however, may exacerbate IPF and increase risk of death while awaiting transplantation. A recent report cited the median waitlist time for lung transplant patients to be 3.7 months, and overall waitlist mortality was 10.4 per 100 patient years [10].

Successful wound healing is a critical aspect of any surgical procedure, but is particularly important in lung transplantation. Airway complications (e.g., anastomotic dehiscence) after lung transplantation are associated with increased morbidity and mortality, and the incidence of such complications has been reported as anywhere from 1.6 to 33% [11, 12]; expert consensus is approximately 15% [12]. The mortality rate from these complications has been reported as 2 to 4% [13]. The incidence of sternal complications with the traditional clamshell incision, which includes sternal dehiscence, has been reported as 34 to 36% [14]. These complications are real threats to the success of a lung transplantation procedure, and so in this study we considered whether the use of pirfenidone pre-transplant would increase the risk of impaired wound healing after lung transplantation.

In this study, “impaired wound healing” was defined as the occurrence of delayed or defective healing of surgical incision, anastomotic dehiscence, or sternal malunion. At Norton Thoracic Institute in Phoenix, Arizona, several patients with IPF have continued treatment with pirfenidone until lung transplantation, allowing us a unique opportunity to identify whether wound healing was impaired in these patients. We analyzed the occurrence of impaired wound healing post-lung transplantation.

## Methods

### Patient selection

This study was approved by the Institutional Review Board at St. Joseph’s Hospital and Medical Center in Phoenix, Arizona. Patients were eligible for the study if they were at least 18 years old, had clinical or histological diagnosis of IPF, and underwent lung transplantation at Norton Thoracic Institute between January 2014 and December 2015. Although pirfenidone was not approved until October 2014, some patients were taking pirfenidone before approval as part of another study, so we were able to include these patients in this analysis. We included all patients who took pirfenidone up to one month before transplantation, as the biological half-life of this drug is unknown [15].

### Study design and data collection

We conducted a retrospective review of each patient’s inpatient hospital records and outpatient clinic charts, both before and after transplant. Inpatient chart review included physician and surgical notes, chest radiographs, computed tomograms, and bronchoscopy operative reports. If a patient took pirfenidone pre-transplant, the duration of use was recorded. Chart review continued until 90 days post-transplant, as most surgical complications occur within this time frame [15].

Confirmation of pirfenidone use and its duration were obtained from physician notes, external prescription history, from the patient during the transplant evaluation interview, and from the medication administration record from patients who were hospitalized pre-transplant. Laboratory data were collected within 24 h before transplantation. Aspartate aminotransferase, alanine aminotransferase, alkaline phosphatase, albumin, bilirubin, and serum creatinine were assessed to determine renal and liver function. Serum glucose and phosphorous levels, and corticosteroid use pre-transplant were also measured to assess for any potential confounding causes of wound healing. Pre-transplant phosphorous levels were unavailable for most patients; this value was collected from the initial post-transplant labs. Pre-transplant medication lists were reviewed for corticosteroids, as corticosteroids are known to delay wound healing. Immunosuppressant regimens post-transplant were also included in analysis. Some patients had a change in immunosuppressive regimen; in these cases, all immunosuppressants taken were included. All patients received induction and triple therapy immunosuppression including tacrolimus, mycophenolate mofetil, and high-dose intravenous steroids, which were tapered down to 10 mg of prednisone daily. Induction regimens were decided prior to transplant. Patients with panel-reactive antibodies received rituximab or thymoglobulin; all other patients received basiliximab induction. All patients who underwent bilateral lung transplant had clamshell incisions; all unilateral lung transplants were performed via thoracotomy.

Evaluation of the anastomotic site was routinely carried out during postoperative bronchoscopies and during scheduled routine surveillance bronchoscopies at 1, 3, 6, and 12 months. Baseline data and wound healing outcomes were also collected in patients diagnosed with IPF who were not taking pirfenidone but underwent transplant during the same time period. The primary outcome of impaired wound healing was defined as the occurrence of delayed or defective healing of surgical incision, anastomotic dehiscence, or sternal malunion. The chest wall incision and anastomotic sites were evaluated to determine the outcome of surgical incisions.

## Results

Out of the 166 patients who underwent lung transplantation between January 2014 and December 2015, 18 received pirfenidone pre-transplantation. Out of those 18 patients, 17 ones had taken pirfenidone up to a month prior to transplantation. Out of these 17 patients, 13 (76.5%) were taking 801 mg of pirfenidone three times daily, while four patients (23.5%) were taking decreased doses after experiencing adverse effects. All patients had been taking pirfenidone for more than 30 days pre-transplant, and ten patients (58.8%) had been taking pirfenidone for more than 90 days. Three patients (17.6%) had been on pirfenidone for several years prior to lung transplantation. Thirteen patients (76.5%) took pirfenidone right up to the time of transplantation. Characteristics of the four patients for whom pirfenidone was discontinued pre-transplantation more than 24 h prior to transplant are summarized in Table 1. Pirfenidone was discontinued in two patients due to initiation of extracorporeal membrane oxygenation (ECMO): one patient due to cost and one patient due to adverse gastrointestinal effects.

**Table 1:** Patients who discontinued pirfenidone more than 1 day prior to transplantation

Variable	Patient 1	Patient 2	Patient 3	Patient 4
Age, years	59	68	67	69
Number of days before LTx pirfenidone was stopped	6	15	16	21
Pirfenidone dose at time of discontinuation	801 mg PO TID	801 mg PO TID	801 mg PO TID	801 mg PO TID
Duration of pirfenidone treatment	30-90 days	> 90 days	30-90 days	< 30 days
Reason for stopping pirfenidone	VV ECMO BTT with feeding tube	ECMO BTT with feeding tube	Cost	Adverse GI effects
Prednisone dose at LTx	10 mg PO daily	10 mg PO daily	20 mg PO daily	None

Abbreviations: *BTT* bridge to transplantation, *GI* gastrointestinal, *LTx* lung transplantation, *PO* per oral, *TID* ter in die (3 times per day), *VV ECMO* venovenous extracorporeal membrane oxygenation

Baseline characteristics and patient outcomes are described in Tables 2 and 3, respectively. All patients had stable renal and liver function, and none were malnourished (based on body mass index). The mean duration of follow-up in the pirfenidone group was 94 days (range, 84–110 days). One patient did develop sternal dehiscence 19 days postoperatively. No patient in the comparator group experienced impaired wound healing.

**Table 2:** Patient characteristics

Variable	Pirfenidone *n* = 17	Control *n* = 13
Demographics		
Mean age, years (range)	67 (60-77)	65 (58-73)
Female sex	2 (11.8)	3 (23.1)
Mean BMI, kg/m^2^ (range)	25.7 (20.45-33.5)	26.9 (20.2-34.6)
ECMO as bridge to transplant	2 (12)	1 (7.7)
Inpatient prior to transplant	6 (35.3)	4 (30.8)
Unilateral lung transplant	3 (17.6)	0 (0)
Mean LAS (range)	46 (32-90)	57 (33-94)
Pre-transplant steroid use	8	7
Prednisone ≤ 10 mg daily	10 (58.8)	6 (46.2)
Prednisone > 10 mg daily	1 (5.9)	1 (7.7)
Baseline laboratory values		
Glucose > 180 mg/dL	0 (0)	0 (0)
ALT > 55 units/L	1 (5.9)	0 (0)
AST > 34 units/L	0 (0)	1 (7.7)
AlkPhos > 150 units/L	0 (0)	0 (0)
Albumin < 3.5 g/dL	2 (11.8)	3 (23.1)
Bilirubin > 1.2 mg/dL	0 (0)	1 (7.7)
Creatinine > 1.25 mg/dL	0 (0)	0 (0)
Phosphorous < 2.4 mg/dL	0 (0)	2 (15.4)
Immunosuppressant therapy post-transplant		
High-dose steroid + taper	17 (100)	13 (100)
Basiliximab	11 (64.7)	8 (61.5)
Tacrolimus	17 (100)	13 (100)
Cyclosporine	0 (0)	2 (15.4)
Mycophenolate	17 (100)	12 (92.3)
Rituximab	4 (23.5)	4 (30.8)
Antithymocyte globulin	2 (11.8)	0 (0)
Azathioprine	0 (0)	1 (7.7)

All values expressed are n (%), unless otherwise indicated

Abbreviations: *ALT* alanine aminotransferase, *AST* aspartate aminotransferase, *BMI* body mass index, *ECMO* extracorporeal membrane oxygenation, *LAS* lung allocation score

**Table 3:** Patient outcomes

Variable	Pirfenidone *n* = 17	Control *n* = 13
Pirfenidone duration pre-transplant		
< 30 days	1	N/A
30-90 days	6 (35.3)	N/A
> 90 days	10 (58.8)	N/A
Pirfenidone regimen		
801 mg TID	13 (76.5)	N/A
534 mg TID	3 (17.6)	N/A
201 mg TID	1 (5.9)	N/A
Mean follow-up, days (range)	94 (84-110)	96 (86-127)
Impaired wound healing	1 (5.9)	0

All values expressed are n (%), unless otherwise indicated

Abbreviations: *N/A* not applicable, *TID* three times per day

## Discussion

Our study corroborates the findings of previous single-center studies: Pirfenidone does not seem to be correlated with increased wound healing complications posttransplant. Two previous reports describing four patients who continued pirfenidone until lung transplantation showed no adverse events posttransplant, but wound healing was not addressed in either publication [16, 17]. In 2014, Riddell et al. described three patients who were bridged to transplant with pirfenidone [16]. Patients spent between 35 and 271 days on the transplant waitlist and had been taking pirfenidone from 190 to 768 days. No adverse events occurred posttransplant; however, pirfenidone doses were not provided, and posttransplant data were not described. In 2015, Paone et al. described a 62-year-old male who started taking pirfenidone after being placed on the transplant list [17]. This patient took 2403 mg of pirfenidone each day for at least 6 months before transplant, and his functional respiratory rate stabilized. He remained on pirfenidone until he underwent unilateral lung transplantation with an ex vivo lung perfusion approach. At 20 months’ follow up, the patient was considered to be in good condition. Another article by Leuschner et al. [15] described the effects of antifibrotic therapy in patients with IPF undergoing lung transplantation. Their series included 23 patients who were taking pirfenidone, seven patients who were taking nintedanib, and 32 control patients. However, 18 patients in their study (29.0%) required posttransplant surgical revisions due to bleeding, impaired wound healing, or both. Out of these 18 patients, 30.4% took pre-transplant pirfenidone, 14.3% took pre-transplant nintedanib, and 31.3% were from the control group.

However, in our series, the sole patient who developed sternal dehiscence was on ECMO as a bridge to transplantation. During repair, it was noticed that all of his intercostal stitches had broken, which caused sternal dehiscence. In addition, the patient was on a very high steroid dose starting at 80 mg/day after his transplant. According to inpatient medication administration records, this patient’s last dose of pirfenidone was taken 15 days before transplantation, so the concentration of pirfenidone was likely minimal at the time of transplantation. We, therefore, classified this as a surgical complication rather than an effect of the pirfenidone.

One patient in our study who underwent unilateral lung transplantation continued taking pirfenidone posttransplant for the pulmonary fibrosis in the native lung. This patient resumed pirfenidone approximately 30 days after transplantation. At last follow up, the patient was 15 months posttransplant, was still taking pirfenidone, and was doing well.

This study is limited by its relatively small sample size. Similar to other retrospective analyses, we were unable to control for all confounding factors and had limited information on compliance. Confirmation of pirfenidone compliance was only available for the 2 patients who were admitted to the hospital pre-transplant.

## Conclusion

Pirfenidone has been shown to successfully diminish the effects of IPF, but lung transplantation remains the treatment of choice for patients with advanced disease. Although pirfenidone inhibits TGF-β, a known contributor to wound healing, patients in this study who continued taking pirfenidone until lung transplantation did not experience impaired wound healing.
